# Cost-effectiveness of West Nile Virus Vaccination

**DOI:** 10.3201/eid1203.050782

**Published:** 2006-03

**Authors:** Armineh Zohrabian, Edward B. Hayes, Lyle R. Petersen

**Affiliations:** * Centers for Disease Control and Prevention, Atlanta, Georgia, USA;; † Centers for Disease Control and Prevention, Fort Collins, Colorado, USA

**Keywords:** West Nile virus, cost-effectiveness, vaccination, perspective

## Abstract

West Nile virus (WNV) was first detected in the Western Hemisphere in 1999 in New York City. From 1999 through 2004, >16,600 cases of WNV-related illnesses were reported in the United States, of which >7,000 were neuroinvasive disease and >600 were fatal. Several approaches are under way to develop a human vaccine. Through simulations and sensitivity analysis that incorporated uncertainties regarding future transmission patterns of WNV and costs of health outcomes, we estimated that the range of values for the cost per case of WNV illness prevented by vaccination was US $20,000–$59,000 (mean $36,000). Cost-effectiveness was most sensitive to changes in the risk for infection, probability of symptomatic illness, and vaccination cost. Analysis indicated that universal vaccination against WNV disease would be unlikely to result in societal monetary savings unless disease incidence increases substantially over what has been seen in the past 6 years.

## Abstract 2

Vaccination is unlikely to result in societal monetary savings.

West Nile virus (WNV) was first detected in the Western Hemisphere in 1999 during an outbreak of encephalitis in New York City (*1*). Over the next 6 years the virus spread across the continental United States, as well as into Canada, Latin America, and the Caribbean islands (*2**,**3*). From 1999 through 2004, >16,600 WNV illnesses in humans have been reported in the United States; >7,000 of these were neuroinvasive disease, and >600 were fatal. In 2002 alone, 2,942 cases of neuroinvasive WNV disease were reported in the United States, which represents the largest epidemic of neuroinvasive WNV disease ever recorded (*4*). Approximately 20% of WNV infections in humans result in symptomatic illness, and ≈1% of infections lead to encephalitis, meningitis, or acute flaccid paralysis (*1*). A substantial proportion of persons in whom severe neuroinvasive WNV disease develops have long-term disability or die as a result of their infection (*5**,**6*).

WNV is transmitted to humans primarily through the bite of infected mosquitoes, but transmission through blood transfusion, through organ donation, and from mother to child have been described (*7*). Strategies to prevent WNV infection include avoiding exposure to infected mosquitoes, reducing the abundance of mosquito vectors, and screening infected blood donations before transfusion. Several approaches are under way to develop a safe and effective human vaccine (*8**–**10*). The public health utility of a new vaccine will depend largely on the incidence, geographic distribution, and severity of WNV disease in the United States, as well as the cost of vaccination. We evaluated the cost-effectiveness of vaccination against WNV in the United States from a societal perspective. Uncertainties regarding the future transmission patterns of WNV and the costs of health outcomes preclude an exact estimation of the economic impact of vaccination. Through probabilistic sensitivity analysis, which incorporates these uncertainties, we estimated the range of most likely values for the cost-effectiveness of vaccination and described the variables that have the most impact on the economic outcome of vaccination. We also estimated the likelihood that a universal vaccination program would result in economic savings.

## Methods

The decision tree used to evaluate the cost-effectiveness of vaccination compared with no vaccination is shown in the Figure. Vaccination was assumed to have no effect on the incidence of infection or the severity of WNV illness but rather to influence only the proportion of infected persons in whom symptoms would develop. Baseline probabilities for each of the chance nodes in the tree were derived by reviewing published articles on the incidence, clinical manifestations, and outcomes of WNV disease as described in further detail below. We estimated the average cost per case of WNV illness prevented, that is, average cost-effectiveness ratio (ACER) (Appendix 1) by calculating the expected societal costs of WNV illness with a vaccination strategy, subtracting the costs of illness with no vaccination, and dividing the remainder by the number of cases prevented by vaccination.

**Figure:**
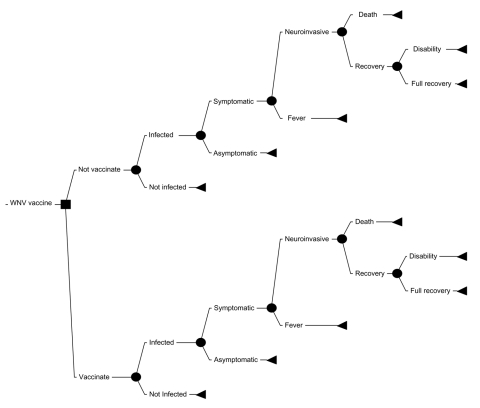
Decision tree for vaccination program. WNV, West Nile virus.

### Time Horizon

We assumed that a single dose of live-attenuated WNV vaccine would provide immunity for >10 years, as is true for the currently licensed yellow fever vaccine (*10**,**11*). If an inactivated vaccine were used, 2 or 3 initial doses would probably be required, and booster doses would probably be needed every 3 years, as is currently recommended for inactivated Japanese encephalitis vaccine (*12*). Both the cost and effectiveness of vaccination were assumed to be the same whether achieved through a single live-attenuated vaccine dose or multiple inactivated vaccine doses at a lower cost per dose.

Although the time horizon for risk for illness, protection from the vaccine, and cost of vaccination was 10 years, we used estimated lifetime costs of disease outcomes in our model. Thus, we modeled the difference in lifetime costs of illness that would be incurred by society during a 10-year period under an immediately implemented universal vaccination strategy compared with no vaccination.

The probabilities of outcomes and costs modeled are average probabilities for the entire population, regardless of age. Our analysis therefore estimates ranges of average societal costs and outcomes prevented when all people in the society are vaccinated, regardless of the age at vaccination or illness. A more detailed analysis of the effect of vaccinating certain age groups would require estimates of age-specific risks and costs of outcomes, which are not readily available for most outcomes in the model.

### Estimation of Costs

The overall cost of WNV illness per person at risk was calculated as the sum of the average costs for each health outcome weighted by the probability of occurrence of each outcome (Table A1). Both medical treatment costs and productivity losses due to illness and death from WNV infection were included in cost estimates. We considered the following health outcomes of WNV infection in our analysis: asymptomatic infection, uncomplicated febrile illness with full recovery, neuroinvasive illness (encephalitis, meningitis, or paralysis) with full recovery, neuroinvasive illness with residual long-term disability, and death.

Asymptomatic infection was assumed to have no cost. Estimates of the cost of uncomplicated febrile illness due to WNV infection were not available so we assumed a cost of US $1,000 per case, based on 5 days of lost productivity at $165 per day (*13*), plus an assumed $175 in medical costs that included 1 ambulatory care visit, diagnostic tests, and outpatient medications. Precision of this cost estimate was not very important since the cost-effectiveness ratio was not sensitive to the changes in this variable. The estimated cost per case of neuroinvasive WNV illness with full recovery ($27,500) was derived from an economic study conducted during the 2002 WNV epidemic in Louisiana (*14*) in which economic costs, rather than charges, were considered a measure of resources. Our goal was to measure the forgone benefits that could have been derived if the resources had been allocated to their next best use, i.e., the opportunity cost. Charges made by healthcare providers do not usually reflect the opportunity costs because of healthcare market imperfections. Charges were adjusted to economic costs through the use of cost-to-charge ratios (for details see Appendix 2 in reference 14; we adjusted 2002 dollars to 2004 dollars, the last year for which consumer price indices were available at the time of this study [15,16]). This cost of neuroinvasive illness included costs of outpatient evaluation, inpatient treatment, rehabilitation treatment, lost productivity of the patient and caregiver at home, and transportation (Table A1). Estimates of the cost of residual long-term disability after neuroinvasive disease were not available, but many of the disabilities that have been described after WNV illness are clinically similar to those that result from acute stroke, and the 2 conditions both affect primarily older males. We therefore used estimates of the lifetime cost of stroke as a proxy for the cost per case of neuroinvasive WNV illness with residual long-term disability (*17*) (1990 dollars adjusted to 2004 dollars [15,16]). Details are shown in Appendix 2.

The average societal cost due to death from WNV disease was estimated by using productivity loss tables (*13*) and the age distribution of 713 WNV nationwide deaths reported to the ArboNET database of the Centers for Disease Control and Prevention (CDC) since 1999 (CDC, unpub. data). The median age of fatal cases was 77 years (range 1 month to 99 years). The estimated cost due to death was $200,000 at a 3% discount rate (2000 dollars from productivity tables [13] were adjusted to 2004 dollars [16]). Since short-term costs in our model were randomly distributed throughout the 10-year time horizon, to simplify the model, we only discounted the long-term costs, such as long-term disability costs and costs due to death. For the short-term costs incurred within the 10-year time horizon, we assumed our estimates represented the present values of those costs (Appendix 2).

Since no human WNV vaccine was licensed at the time of our evaluation, vaccine costs were not available. Based on charges in the United States for yellow fever vaccine (≈$85 per dose), hepatitis A vaccine (≈$75 per dose), Japanese encephalitis vaccine (≈$315 for a 3-dose series), and the previously available Lyme disease vaccine (≈$150 for a 3-dose series), we assumed a total baseline vaccination cost of $100 to include both the actual cost of the vaccine and the cost of administering the vaccine. For the sensitivity analysis focused on the cost of vaccination, we assumed minimum and maximum vaccination costs of $10 and $150, respectively (see below).

### Estimates of Probabilities for Health Outcomes

#### Probability of Infection

Several seroepidemiologic surveys have estimated the proportion of North American populations who were infected with WNV during epidemic transmission. The highest seroprevalence published to date is 2.6% (*1*). In 2002, during the largest epidemic of WNV neuroinvasive disease ever described in the United States, 2,942 neuroinvasive WNV disease cases were reported from 36 states and the District of Columbia (total population ≈253.4 million). If one assumes a ratio of 1 neuroinvasive case for every 140 infections, which was the finding of a 1999 household-based seroepidemiologic survey in New York City (*1*), this yields an overall estimate of ≈411,880 infections and an estimated incidence of 0.16 infections per 100 people, or 0.0016 per person per year. Whether WNV epidemics will continue to occur in the United States at a similar frequency or intensity is unknown, but for this analysis we assumed that the risk for WNV infection would be 0.0016 per person per year for 10 years. The cumulative risk for WNV infection over a 10-year period would be 1 – e^(–0.0016 × 10)^ = 0.016. We therefore estimated the baseline probability of infection as 0.016. For sensitivity analysis focused on probability of infection, we assumed for the minimum risk for infection that a person would encounter only 1 year of WNV transmission, yielding a cumulative risk of 0.0016 over the 10-year period. For the maximum risk, we assumed that the risk would be that of yearly epidemic transmission such that 2.6% of the population would be infected each year over the 10-year period, yielding a 10-year cumulative risk of 0.23. Further details regarding sensitivity analysis are described below.

#### Probability of Symptomatic Illness and Vaccine Effectiveness

We assumed that symptoms of WNV illness will develop in 20% of infected persons and that neuroinvasive disease will develop in 3.6% of them, which is equivalent to 1 neuroinvasive case for every 140 infections previously described (*1*). We also assumed a vaccine effectiveness of 80% in reducing the risk for symptomatic illness.

#### Probability of Long-term Disability or Death after Neuroinvasive WNV Disease

Precise data on long-term outcomes from WNV illness are limited. A study of 19 patients with neuroinvasive WNV disease found that 2 (11%) died, and of the 17 survivors, 7 (41%) had recovered fully at the time of discharge, 6 (31%) were discharged without full recovery, and 4 (24%) were discharged to a long-term care facility (*18*). Another study of 57 patients with neuroinvasive disease found that 10 (18%) eventually died, 13 (23%) were discharged without support, 14 (25%) were discharged requiring support, 14 (25%) were discharged to a rehabilitation facility or nursing home, 4 (7%) moved in with relatives, and 2 (4%) remained in an acute care facility (*5*). A study of 16 patients with neuroinvasive WNV disease found that 1 patient (6%) died and that 8 months after illness, 4 (25%) patients required assistance or rehabilitation and 11 (69%) were functioning independently at home (*19*). A survey of 35 patients who had been hospitalized with WNV illness found that 63% reported full recovery 12 months after illness onset (*6*). Based on the limited data from these studies, we assumed that 35% of patients would have lifelong disability after neuroinvasive WNV disease. Of 2,942 patients with neuroinvasive WNV disease reported in the United States in 2002, 276 (9%) died (*4*). For our model, we assumed a case-fatality ratio of 9%.

### Sensitivity Analysis

To incorporate uncertainties regarding the values of all input variables, we assigned uniform probability distribution to all variables, allowing 25% variability around the baseline values (Table 1). We used @Risk Analysis 2002 software (Palisade Corporation, Newfield, NY, USA) to generate distributions of possible outcomes by Monte Carlo simulation of the ACER using 5,000 iterations that covered all combinations of input variable values. The results provided detailed summary statistics for the ACER distribution, including the 5th and 95th percentile ranges of values and the probability that vaccination would result in societal savings. To further investigate the impact of the risk for infection and vaccination cost on the ACER, we ran separate simulations in which the minimum, baseline, and maximum values for these variables described in the corresponding sections were fixed, while all other variables were allowed to vary according to their prespecified uniform distributions.

**Table 1:** Uniform distributions for each variable used in simulations to assess the cost-effectiveness of vaccination against West Nile virus (WNV)*

Variable	Lower limit	Baseline	Upper limit
Probability of infection	0.012	0.016	0.02
Probability of symptomatic illness	0.15	0.20	0.25
Probability of symptomatic illness after vaccination†	0.03	0.04	0.05
Probability of neuroinvasive disease, given symptoms	0.027	0.036	0.045
Probability of death, given neuroinvasive disease	0.07	0.09	0.11
Probability of disability, given neuroinvasive disease	0.26	0.35	0.44
Cost of neuroinvasive disease	$20,625	$27,500	$34,375
Cost of death (direct and indirect financial losses)	$150,000	$200,000	$250,000
Cost of lifelong disability	$158,000	$210,000	$263,000
Cost of uncomplicated WNV febrile illness	$750	$1,000	$1,250
Cost of vaccination	$75	$100	$125

*Upper and lower limits are calculated as ±25% of the baseline values and rounded up. †Baseline vaccine effectiveness is assumed to be 80%.

## Results

Using baseline values of all input variables, without accounting for uncertainties, the average cost per case of WNV illness prevented would be ≈$34,200. At a cost of $8.7 billion in a hypothetical population of 100 million people, vaccination would prevent 256,000 cases of WNV illness, including 9,216 cases of neuroinvasive disease, 2,935 cases of lifetime disability, and 829 deaths during a 10-year period. Under these assumptions, universal vaccination would yield societal savings if the cumulative incidence of WNV infection over a 10-year period were >0.13 (≈1.4% of the population infected each year), the cost of vaccination were <$12.8, or the cost of lifelong disability were >$3.2 million (≈15 times higher than the baseline estimate).

The simulation results accounting for uncertainties in all input variables are shown in Table 2. The median of the ACER distribution was $35,000 per case of WNV illness prevented. The 5th and 95th percentiles for the ACER were $59,000 and $20,000, respectively.

**Table 2:** Outcome distributions of average cost-effectiveness ratio (ACER) accounting for variability in all input variables*

Statistic	ACER†
5th–95th percentile range, $	–59,000 to –20,000
Mean, $	–36,000
Median, $	–35,000
Mode, $	–33,000
Probability of savings, %	0

*According to the distribution provided in Table 1. †Negative value indicates cost.

To identify the sensitivity of the output to all input distributions, we used @Risk sensitivity analysis with a regression in which the dependent variable was the output variable, i.e., ACER, and the independent variables were the input variables presented as @Risk uniform distribution functions (Table 1). Each iteration represented an observation for the regression. The coefficients calculated for each input variable measured the sensitivity of the output to that particular input distribution. The results indicated that ACER was most sensitive to the changes in the risk for infection, probability of symptomatic illness, and vaccination cost (Table 3). A 1 standard deviation (SD) increase in the probability of symptomatic illness increased the ACER by an SD of 0.65, while a 1 SD increase in the probability of infection or the vaccination cost increased the ACER by an SD of 0.5. Changes in the other variables had little or no impact on ACER (Table 3).

**Table 3:** Sensitivity of the average cost-effectiveness ratio (ACER) for input variables

Rank	Input variables	Regression coefficient†
1	Probability of symptomatic illness	0.65
2	Probability of infection	0.51
3	Vaccination cost	0.50
4	Probability of symptomatic illness after vaccination	–0.14
5	Probability of neuroinvasive disease, given symptoms	0.05
6	Cost of lifelong disability	–0.03
7	Probability of disability, given neuroinvasive disease	0.03
8	Cost of neuroinvasive disease	–0.02
9	Cost of uncomplicated WNV febrile illness*	–0.01
10	Cost of death (direct and indirect financial losses)	–0.01
11	Probability of death, given neuroinvasive disease	0.00

*WNV, West Nile virus. †@Risk analysis software runs a regression where the dependent variable is the output variable, i.e., ACER, and the independent variables are the input variables presented as @Risk uniform distribution functions. Each iteration represents an observation for the regression. The coefficient calculated for each input variable measures the sensitivity of the output to that particular input distribution. For example, a coefficient of 0.65 indicates that a 1–standard deviation (SD) increase in probability of symptomatic illness increases the ACER by an SD of 0.65.

The results of the sensitivity analysis focused separately on risk for infection and vaccination cost are shown in Table 4. The probability that vaccination would yield societal savings changed from 0% to 98% when the 10-year cumulative risk for WNV infection changed from 0.016 to 0.230, and from 0% to 76% as the vaccination cost decreased from $150 to $10.

**Table 4:** Sensitivity of the average cost-effectiveness ratio for stepwise changes in infection rate and vaccination cost*

Statistic	Infection rate			Vaccination cost		
	0.0016	0.016	0.23	–150	–100	–10
5th–95th percentile range, $	–585,000 to –261,000	–54,000 to –22,000	343 to 3,846	–86,000 to –36,000	–56,000 to –23,000	–1,400 to 2,900
Mean, $	–400,000	–36,000	2,096	–57,000	–36,000	860
Median, $	–386,000	–34,000	2,098	–55,000	–35,000	920
Mode, $	–373,000	–30,000	1,500	–54,000	–36,000	740
Probability of savings, %	0	0	98	0	0	76

*All other variables were allowed to vary according to their specified uniform distributions provided in Table 1. Negative value indicates cost; positive value indicates monetary saving.

## Discussion

The economic impact of a vaccination strategy is a determinant of the public health decision regarding whether or not to recommend vaccination, but it is certainly not the only determinant of sound public health vaccination policy. It is also not imperative that a vaccination program result in monetary savings for it to be cost-effective compared with other public health interventions. Societies and people are willing to pay for preventing disease, as indicated by the implementation of preventive interventions that do not result in economic savings, and most relevant, the willingness to pay for expensive vaccines (*20**–**22*). However, as public health implications of vaccination programs are considered, we must have some understanding of the resources that might be expended. Vaccination would be most appealing if it is likely to safely prevent disease and save society money, or at least have a relatively low cost per case of illness prevented.

Our analysis indicates that a universal vaccination program to prevent WNV disease would be unlikely to result in societal monetary savings unless the incidence of the disease increases substantially over what has been seen in the past 6 years, or the cost of vaccination were <$12 per person vaccinated. The risk for WNV infection, probability of symptomatic illness after infection, and cost of vaccine appeared to have the greatest influence on the cost-effectiveness outcome. Within the range of possible values used in our model, variations in vaccine effectiveness, cost of WNV illness, and probabilities of various health outcomes did not lead to considerable change in the cost-effectiveness.

The future patterns of WNV transmission in North America cannot be accurately predicted. The virus was first detected in North America in 1999, and the epidemiology of WNV illness in the Western Hemisphere continues to evolve. The antigenically related flaviviruses St. Louis encephalitis virus (SLEV) and Japanese encephalitis virus (JEV) demonstrate different patterns of transmission that WNV could assume; SLEV is sporadically transmitted in North America with intense epidemics separated by years of low-level transmission, while JEV occurs in Asia with annual epidemics of intense transmission. If WNV assumes a transmission pattern in North America similar to that of JEV in Asia, then vaccination is likely to be a much more appealing public health prevention strategy and is likely to be more cost-effective than if WNV transmission follows the pattern of SLEV. As WNV spreads southward into Latin America, increased incidence may be seen with less protection from mosquitoes provided by air conditioning and screens (*23*). If intense transmission is seen in these areas, vaccination may be the most cost-effective prevention strategy, but unless the vaccine cost is low, it may still be too expensive for local economies.

WNV infection may cause severe untreatable neurologic disease. While the risk is highest in the elderly, severe disease does occur among young adults and children (*4**,**24*). The more severe, untreatable manifestations of WNV infection would compel interest in vaccine development and use even if vaccination is expensive, particularly since current prevention strategies such as personal repellent use or environmental reduction of mosquito abundance may not be consistently implemented (*25*). The effectiveness of these other prevention strategies is difficult to conclusively demonstrate and estimates of their cost-effectiveness have not been published. Vaccination may reduce the expenditures for mosquito control in certain areas, but we did not include this possible effect in our model. If alternate prevention costs were reduced by vaccination, we would expect this to improve the cost-effectiveness of vaccination from the societal perspective.

Our results provide a general assessment of the likely economic implications of universal vaccination against WNV and an indication of which parameters have the greatest influence on the cost-effectiveness of vaccination. A safe and effective vaccine may prove to be the most effective, and perhaps the most cost-effective, strategy to prevent severe WNV illness. The economic impact of vaccination will depend mostly on the risk for WNV infection, probability of symptomatic illness after infection, and the cost of vaccination.

## Appendix 1

### Cost-effectiveness Formula

We used the following formula to calculate the cost per case of West Nile virus (WNV) illness prevented:


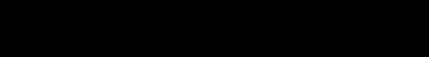
,

where *ACER* is the average cost-effectiveness ratio, N is the size of the population at risk for WNV illness, C_V_ is the cost of vaccination, and 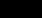 and 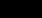 are the expected costs of a case of WNV illness without and with vaccination estimated as the weighted average cost of all health outcomes (the health outcomes and their corresponding costs and probabilities are presented in the Table A1). The denominator is the expected number of cases prevented due to vaccination.

The overall sign of ACER is determined by the numerator. The denominator is always positive because the expected number of symptomatic case-patients without vaccination, 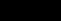, is always higher than the expected number of symptomatic cases with vaccination, 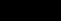. When vaccination costs are less than the expected savings due to vaccination, the numerator and ACER will be positive, which indicates net savings due to vaccination.

## Appendix 2

### Discounting, Lifetime Disability Costs, and Costs due to Death

Discounting is an economic notion that even in a world of zero inflation, a dollar today would be of higher value to a person than a dollar in the future. The premium placed on benefits today versus the future is reflected in the rate at which a person is willing to exchange present for future costs and benefits. This quantitative measure of time preference is called the *discount rate*. When the costs or benefits under the study continue in the future, to make them comparable in terms of the time dimension economists calculate the present value of these costs or benefits by using discount rates. Different discount rates have been used in the literature: conceptually the appropriate discount rate depends on the perspective of the study and the question it poses. The US Public Health Service Panel on Cost-effectiveness in Health and Medicine recommends a 3% discount rate for economic studies in health (*26*).

The average societal cost due to death from West Nile virus (WNV) disease was estimated by using productivity loss tables (*27*) (we adjusted the costs from 2000 dollars to 2004 dollars) and the age distribution of 713 WNV nationwide deaths reported to the ArboNET database of the Centers for Disease Control and Prevention (CDC) since 1999 (CDC 2005, unpub. data). We estimated these costs both at a 5% and a 3% discount rates and, following the recommendations by the US Public Health Service Panel on Cost-effectiveness in Health and Medicine (*26*), we used the 3% discount rate estimate in our model, which yielded a death cost of $200,000 in 2004 dollars (at a 5% discount rate the death cost was $170,000 in 2004 dollars).

As a proxy for lifetime disability costs due to WNV illness, because of insufficient data, we used the lifetime costs of stroke available from the literature (*28*). Although the disability cost of stroke may underestimate the disability cost due to WNV because the median age for WNV neuroinvasive patients is lower (64 years of age [29]) than the median age of persons disabled due to stroke (76 years of age [30]), this will not have significant effect on the results because the cost-effectiveness ratio was not sensitive to changes in the cost of disability. The estimate of the cost of disability due to stroke in 1990 dollars was discounted at a 5% rate. We converted this estimate to 2004 dollars by using the Consumer Price Index for medical care (*31*) and the average hourly earnings of production workers (*32*). This estimate was ≈$180,000 in 2004 dollars.

To make the lifetime disability costs and the death costs comparable, we used the ratio of the 3% discounted death cost ($200,000) and the 5% discounted death cost ($170,000) as a multiplier for adjusting the disability cost discounted at a 5% ($180,000) to a disability cost discounted at 3%. The result was $210,000 in 2004 dollars, which we used as an estimate for a 3% discounted disability cost.

## Appendix

**Table A1:** Cost components and probabilities of incurring a given cost for a patient with neuroinvasive West Nile virus illness used to estimate weighted average cost of illness

Cost category	Average values in 2004 dollars*	Probability of incurring given cost†
Inpatient treatment	19,197	1.00
Inpatient rehabilitation treatment	14,977	0.14
Outpatient hospital cost	316	0.32
Outpatient medical visits	426	1.00
Outpatient physical rehabilitation	3,859	0.22
Outpatient occupational rehabilitation	3,822	0.07
Outpatient speech therapy	556	0.01
Nursing home	8,113	0.04
Productivity losses, temporary	8,442‡	0.40
Productivity losses, caregiver	2,406	0.26
Transportation cost	64	1.00
Miscellaneous	1,534	0.14

*Estimated from Zohrabian et al. (*14*). These estimates were in 2002 dollars and adjusted for inflation for 2004, i.e., the latest year for which the appropriate consumer price indices were available. †Relative frequencies estimated from Zohrabian et al. (*14*). ‡Average number of days missed (estimated from Zohrabian et al. [14]) multiplied by the national average cost of a missed workday, including salary, loss of household services, fringe benefits, and payroll taxes.
